# Discovery and mode of action of a novel analgesic β-toxin from the African spider *Ceratogyrus darlingi*

**DOI:** 10.1371/journal.pone.0182848

**Published:** 2017-09-07

**Authors:** Silmara R. Sousa, Joshua S. Wingerd, Andreas Brust, Christopher Bladen, Lotten Ragnarsson, Volker Herzig, Jennifer R. Deuis, Sebastien Dutertre, Irina Vetter, Gerald W. Zamponi, Glenn F. King, Paul F. Alewood, Richard J. Lewis

**Affiliations:** 1 IMB Centre for Pain Research, Institute for Molecular Bioscience, The University of Queensland, Brisbane, Australia; 2 Department of Physiology and Pharmacology, Hotchkiss Brain Institute and Alberta Children’s Hospital Research Institute, The University of Calgary, Calgary, Canada; 3 Institut des Biomolécules Max Mousseron, Université Montpellier - CNRS, Montpellier, France; 4 School of Pharmacy, The University of Queensland, Brisbane, Australia; Indiana University School of Medicine, UNITED STATES

## Abstract

Spider venoms are rich sources of peptidic ion channel modulators with important therapeutical potential. We screened a panel of 60 spider venoms to find modulators of ion channels involved in pain transmission. We isolated, synthesized and pharmacologically characterized Cd1a, a novel peptide from the venom of the spider *Ceratogyrus darlingi*. Cd1a reversibly paralysed sheep blowflies (PD_50_ of 1318 pmol/g) and inhibited human Ca_v_2.2 (IC_50_ 2.6 μM) but not Ca_v_1.3 or Ca_v_3.1 (IC_50_ > 30 μM) in fluorimetric assays. In patch-clamp electrophysiological assays Cd1a inhibited rat Ca_v_2.2 with similar potency (IC_50_ 3 μM) without influencing the voltage dependence of Ca_v_2.2 activation gating, suggesting that Cd1a doesn’t act on Ca_v_2.2 as a classical gating modifier toxin. The Cd1a binding site on Ca_v_2.2 did not overlap with that of the pore blocker ω-conotoxin GVIA, but its activity at Ca_v_2.2-mutant indicated that Cd1a shares some molecular determinants with GVIA and MVIIA, localized near the pore region. Cd1a also inhibited human Na_v_1.1–1.2 and Na_v_1.7–1.8 (IC_50_ 0.1–6.9 μM) but not Na_v_1.3–1.6 (IC_50_ > 30 μM) in fluorimetric assays. In patch-clamp assays, Cd1a strongly inhibited human Na_v_1.7 (IC_50_ 16 nM) and produced a 29 mV depolarising shift in Na_v_1.7 voltage dependence of activation. Cd1a (400 pmol) fully reversed Na_v_1.7-evoked pain behaviours in mice without producing side effects. In conclusion, Cd1a inhibited two anti-nociceptive targets, appearing to interfere with Ca_v_2.2 inactivation gating, associated with the Ca_v_2.2 α-subunit pore, while altering the activation gating of Na_v_1.7. Cd1a was inactive at some of the Na_v_ and Ca_v_ channels expressed in skeletal and cardiac muscles and nodes of Ranvier, apparently contributing to the lack of side effects at efficacious doses, and suggesting potential as a lead for development of peripheral pain treatments.

## Introduction

Voltage-gated ion channels, including calcium (Ca_v_) and sodium (Na_v_) channels, are membrane proteins involved in the generation of electrical signals underlying the propagation of action potentials in excitable cells. Ca_v_ and Na_v_ are structurally similar, consisting of a pore-forming α subunit and 1–3 auxiliary subunits, including a number of Na_v_ β isoforms and Ca_v_ β, α_2_δ, and γ auxiliary subunits [1, 2]. The Ca_v_ and Na_v_ α-subunits are large transmembrane proteins (~200–260 kDa) organized into four homologous domains (DI—IV). Each domain comprises six transmembrane α helices segments (S1–S6), with the pore-forming P loop located between the S5 and S6 segments [3]. Each voltage sensor domain spans from S1–S4, with the positively charged residues in S4 serving as gating charges [4]. Nine Na_v_ α subunits (Na_v_1.1–Na_v_1.9) and ten Ca_v_ α subunits (Ca_v_1.1–1.4, Ca_v_2.1–2.3 and Ca_v_3.1–3.3) have been functionally characterized and can be differentiated by their primary structure, current kinetics and relative sensitivity to neurotoxins and small molecules [4].

Animal venoms have been pivotal tools in determining Ca_v_ and Na_v_ structure and physiological function [4,5], providing peptides that either occlude the pore of the channel or influence its gating. Moreover, physiological and pharmacological studies have demonstrated that Ca_v_ channels, including Ca_v_2.2 and a number of Na_v_ channels, including Na_v_1.7, are involved in nociceptive signalling, playing a critical role in the development of chronic pain associated with tissue and nerve injury [6,7, 8]. Importantly, a number of neuropeptides have shown therapeutic potential due to ion channel activity [9], including ω-conotoxin MVIIA isolated from the venom of the cone snail *Conus magus*, which potently and selectively blocks Ca_v_2.2 in pain fibres [6,10]. MVIIA is currently marketed as Prialt for the treatment of severe chronic neuropathic, cancer and HIV/AIDS pain [11, 12], validating Ca_v_2.2 as an important analgesic target. Loss-of-function mutations in the human gene *SCN9A* encoding the Na_v_1.7 α subunit result in a congenital inability to experience pain [13,14], while gain-of-function mutations in *SCN9A* lead to inherited erythromelalgia [15] and paroxysmal extreme pain disorder [16]. Thus, Na_v_1.7 has become an important analgesic target, and numerous spider-venom peptides have been reported that modulate the activity of this channel [17].

Spider venoms are rich sources of peptidic Ca_v_ and Na_v_ channel modulators [18, 19, 20]. In the search for novel analgesic peptides, we used Ca^2+^-based fluorescence assays to guide the isolation of a peptide termed Cd1a, from the venom of the African rear-horned baboon tarantula *Ceratogyrus darlingi*. Functional characterization of Cd1a revealed moderate insecticidal effects against sheep blowflies, modest activity at Ca_v_2.2 channels and potent activity at Na_v_1.7, as well as the Cd1a mode of action on both channels. Cd1a reversed spontaneous pain behaviours induced in mice by activation of Na_v_1.7, demonstrating its analgesic potential.

## Materials and methods

### Spider venom screening and assay-guided isolation of Cd1a

Spider venoms were collected from adult specimens of the family Theraphosidae, Hexathelidae, Desidae, Araneidae, Sparassidae, Pisauridae, Nephilidae and Ctenidae (see supporting information: S1 Table) using electrical stimulation (12 V) applied to the basal part of the chelicerae. The crude lyophilized venoms were dissolved in distilled water, centrifuged (20,000 × g for 5 min) to remove particulates, then the supernatant was stored at −20°C until use.

The lyophilized venoms were diluted in phosphate salt solution (PSS) containing (in mM): NaCl 140, glucose 11.5, KCl 5.9, MgCl_2_ 1.4, NaH_2_PO_4_ 1.2, NaHCO_3_ 5, CaCl_2_ 1.8, 2-[4-(2-hydroxyethyl)piperazin-1-yl]ethanesulfonic acid; HEPES 10), plus 0.1% BSA and nifedipine (10 μM) to fully inhibit endogenous Ca_v_1 channels. The crude venoms (at 40 μg/well) were tested for activity against human (h) Ca_v_2.2 channels in SH-SY5Y cells using a FLIPR^TETRA^ (**Fl**uorimetric **I**maging **P**late **R**eader, Molecular Devices, California, USA) Ca^2+^ imaging assay, as detailed below [21]. Venoms were re-tested at 4 μg/mL to identify the most active venoms. *C*. *darlingi* venom produced complete block of Ca_v_2.2-mediated Ca^2+^ responses at both 4 and 40 μg/well and it was selected for further fractionation to isolate the active peptide.

*C*. *darlingi* venom (0.5 mg) was diluted in 100 μL of solvent A (99.9% trifluoroacetic acid (TFA)/0.1% H_2_O) and fractionated on a reversed-phase high-performance liquid chromatography (RP-HPLC) analytical column (Vydac C18, 250 × 4.6 mm, 5 μm, Grace DDS, IL, USA). Elution was performed with solvent A mixed with a linear gradient of solvent B (90% acetonitrile (ACN)/0.1% TFA in H_2_0) at 5% for 5 min, 5–20% over 5 min, 20–40% over 40 min, and 80% from 50–60 min. Fractions were collected and ~10% of each fraction lyophilized, re-suspended in assay buffer, plated and tested for activity using the FLIPR assay.

Automated protein sequencing was performed by the Australian Proteome Research Facility (Sydney, NSW, Australia), using an Applied Biosystems 494 Procise Protein Sequencing System. Briefly, the purified peptide (Cd1a) was dissolved in 25 mM ammonium bicarbonate, pH 8.0 and reduced with DTT at 56°C for 0.5 h, then alkylated using iodoacetamide at room temperature for 0.5 h. The reduced/alkylated Cd1a was then purified using RP-HPLC (using a Zorbax 300SB-C18 column 3 × 150 mm). A single major peak eluting at 18.3 min was collected, evaporated to 50 μL and loaded onto a Precycled Bioprene-treated disc for Edman sequencing.

### Chemical synthesis

Chemical synthesis of Cd1a was performed using solid phase peptide synthesis (SPPS). Briefly, Cd1a was assembled using standard *in situ* neutralization Boc-SPPS [22] on a 1,4-bis(ethenyl)benzene;(4-methylphenyl)-phenylmethanamine;styrene;hydrochloride (MBHA; Novabiochem, Merck Millipore, Kilsyth, Vic, Australia) resin, employing [benzotriazol-1-yloxy(dimethylamino)methylidene]-dimethylazanium;N-ethyl-N-propan-2-ylpropan-2amine; hexafluorophosphate (HBTU DIEA; Sigma-Aldrich/Auspep P/L Melbourne, Australia) activation. The following side chain protected Boc-L-amino acids were used: Arg(Tos), Asn(Xan), Asp(Chxl), Cys(4-MeBzl), Gln(Xan), Tyr(2-BrZ), Thr(Bzl), Trp(For), Ser(Bzl), Lys(2-ClZ) (Novabiochem, Merck Millipore). The resin was cleaved with hydrogen fluoride (BOC Gases, Sydney, NSW, Australia) for 1 h at 0°C employing *p*-cresol/*p*-thio-cresol scavenger (5% each, Sigma Aldrich). Crude peptide was precipitated from ether, filtered and lyophilized from ACN/H_2_O (Sigma Aldrich). After HPLC fractionation (Column: Vydac C18, 250 × 21 mm; gradient: 5–60% ACN over 60 min), pure reduced peptide (20 mg) at a concentration of 0.2 mg/mL was oxidized overnight at pH 7.8 in a solution of 0.3 M NH_4_OAc/0.3 M guanidine-HCl, in the presence of reduced (GSH) and oxidized (GSSG) glutathione (GSH/GSSG; 100:10 mol. eq.). The most prominent isomer was obtained after RP-HPLC isolation (Column: Vydac C18, 250 × 10 mm; gradient: 5–60% ACN over 60 min) in quantities of 1–2 mg. The purity and identity of this product was confirmed by co-elution with a native sample of Cd1a using liquid chromatography-mass spectrometry (LC-MS).

### Cell culture and fluorimetric assays

SH-SY5Y cells were maintained in Roswell Park Memorial Institute (RPMI) medium supplemented with 10% foetal bovine serum (FBS) and 2 mM L-glutamine at 37°C in a 5% humidified CO_2_ incubator. HEK293 cells stably expressing hNa_v_1.1–1.8 channels α-subunits co-expressed with the Na_v_ β_1_ subunit (Scottish Biomedical, Glasgow, UK; except Na_v_1.6, which was generated by GlaxoSmithKline, Stevenage, UK) were maintained at 37°C in a 5% humidified CO_2_ incubator in Dulbecco's Modified Eagle Medium (DMEM; Invitrogen, MA, USA) supplemented with 10% FBS, 2 mM L-glutamine, and kept under selection antibiotics as per manufacturer’s protocol. Cells were plated 48 h prior to assay at a density of 30,000–50,000 cells/well on 384-well black-walled imaging plates (Corning, NY, USA). All the cells were used up to maximum 20 passages.

The FLIPR assays were performed based on previously described protocols [17, 21, 23]. For the FLIPR calcium-based assays, SH-SY5Y cells were pre-incubated for 30 min (37°C in a humidified 5% CO_2_ incubator) with a Calcium 4 fluorescence dye (Molecular Devices) diluted in PSS buffer + 0.1% BSA and nifedipine (10 μM) when testing for hCa_v_2.2 activity. Conversely, to test for Ca_v_1 activity, cells were pre-incubated with ω-conotoxin CVID (1 μM) for 30 min. After the incubation period the plates were transferred to the FLIPR and camera gain and intensity adjusted for each plate to yield 800–1000 arbitrary fluorescence units (AFU) baseline fluorescence. Ca^2+^ responses were measured using a cooled CCD camera with excitation at 470–495 nM and emission at 515–575 nM. Ten baseline fluorescence readings were taken prior to addition of crude venoms, venom fractions or purified peptides diluted in PSS + 0.1% BSA, then fluorescence readings were taken every second for 300 s. After 300 s, activators were added to the cells and fluorescence readings recorded each second for a further 300 s. Endogenous hCa_v_ channels were activated with 90 mM KCl/5 mM CaCl_2_.

The FLIPR membrane potential assays for sodium channels were performed using HEK293 cells expressing hNa_v_1.1–1.8. The cells were loaded with Membrane Potential Assay Kit Red (Molecular Devices) reconstituted in PSS + 0.1% BSA and incubated at 37°C for 30 min before transfer to the FLIPR. After ten baseline fluorescence readings, changes in fluorescence (excitation 510–545 nm; emission 565–625 nm) in response to addition of antagonists (crude venoms or peptides) were measured every second for 300 s. After 300 s, activators (Na_v_1.8: deltamethrin at 100 μM, Sigma-Aldrich; Na_v_1.6: veratridine at 20 μM, Abcam, Melbourne, VIC, Australia; and all other Na_v_ isoforms: veratridine 70 μM) were then added and the responses monitored for a further 300 s, except for Na_v_1.8 cells, where responses were measured for 1800 s.

A four-parameter Hill equation was used to fit concentration-response curves by nonlinear regression analysis (GraphPad Prism v5.0, San Diego, CA, USA). Results are presented as the mean ± standard error of the mean (SEM) of 3–6 replicates on 384-well plates for each independent experiment, performed 3–6 times. Statistical significance was determined using analysis of variance (ANOVA) or a Student’s *t*-test.

### [^125^I]-GVIA binding assay

SH-SY5Y cell membranes were prepared using an adaptation of the method of Wagner *et al* [24]. The cells were harvested using trypsin/ethylenediaminetetraacetic acid (EDTA; Lonza, Basel, Switzerland), washed once with Dulbecco's phosphate-buffered saline (DPBS; Sigma-Aldrich) and centrifuged for 4 min at 500 × g. After centrifugation, the supernatant was discarded, then the pellet was re-suspended in 10 mL binding assay buffer (20 mM HEPES, 75 mM NaCl, 0.2 mM EDTA and 0.2 mM 2-[2-[2-[2-[bis(carboxymethyl)amino]ethoxy]ethoxy]ethyl-(carboxymethyl)amino]acetic acid; EGTA, adjusted to pH 7.2) containing complete protease inhibitor (Roche Diagnostics, NSW, Australia) and sonicated. The homogenate was then centrifuged at 4°C for 30 min at 40,000 × g, the supernatant discarded, and the pellet dissolved in binding assay buffer containing 10% glycerol, before storage at –80°C prior to use. A bicinchoninic acid (BCA) assay kit (Thermo Scientific, Waltham, MA, USA) was used for protein quantification as per manufacturer’s instructions.

Radiolabelled conotoxin GVIA ([^125^I]-GVIA; iodinated at Tyr22) was prepared using IODOGEN as previously described [25], or purchased (Perkin Elmer, USA), and stored at 4°C for use within 20 days. On the day of the assay, the membranes were thawed on ice and reconstituted to 10 μg/50 μL (mouse) or 10–20 μg/50 μL (SH-SY5Y) in binding assay buffer containing 2% complete protease inhibitor and 0.1% BSA. Stock [^125^I]-GVIA was diluted to 20,000 cpm/50 μL ([30 pM]). For displacement studies, [^125^I]-GVIA was incubated with mouse brain membranes or SH-SY5Y membranes and varying concentrations of the competing ligand on 96-well plates. The plates were incubated with shaking for 1 h at room temperature and vacuum filtered through a glass-fibre filter pre-soaked in 0.6% polyethyleneimine (PEI) to reduce non-specific binding and washed with buffer containing 20 mM HEPES and 125 mM NaCl at pH 7.2 using a Tomtec harvester vacuum system (CT, USA). The filters were then dried at 37°C before being placed in sample bags and soaked in liquid scintillant. Retained radioactivity was then counted using a MicroBeta JET microplate liquid scintillation counter (Wallac, Finland). Non-specific binding was determined in the presence of 50 μL of unlabelled peptides. Specific binding was calculated as the difference between total and non-specific binding. A one-site model was fitted to the data using GraphPad Prism v5.0. Results are presented as the mean ± SEM of 3–6 replicates, performed at minimum 3 independent experiments. Statistical significance was determined using analysis of variance (ANOVA) or a Student’s *t*-test, with statistical significance defined as p < 0.05.

### Electrophysiological properties of rCa_v_2.2 channels

HEK tsA-201 cells used for the Ca_v_2.2 patch-clamp experiments were cultured and transiently transfected with native rat (r) Ca_v_2.2 (Ca_v_ α_1b_ co-expressed with Ca_v_ β_1b_ and Ca_v_ α_2_δ subunits) or mutant rCa_v_2.2-G1326P, as previously described [26]. Transfected cells were incubated for 48 h at 37°C and 5% CO_2_, re-suspended with 0.25% (w/v) trypsin-EDTA (Invitrogen) and plated onto glass coverslips at least 3–4 h before the patch clamp experiments. Ca_v_ currents were measured by conventional whole-cell patch clamp using an Axopatch 200B amplifier in combination with Clampex 9.2 software (Molecular Devices), as previously described [27]. Cd1a was prepared daily in external solution containing 0.1% BSA and applied to the cells with a gravity-driven micro-perfusion system. The external recording solution for calcium channel recordings contained (in mM): 114 CsCl, 20 BaCl_2_, 1 MgCl_2_, 10 HEPES 10 glucose, adjusted to pH 7.4 with CsOH. For voltage-clamp recordings, 5 μM CdCl_2_ was also added to the external solution to inhibit Ca_v_ channels. For all recordings, the internal patch pipette solution contained (in mM): 108 CsMeSO_4_, 2 MgCl_2_, 11 EGTA, 10 HEPES, adjusted to pH 7.4 with CsOH supplemented with 0.6 mM GTP and 2 mM ATP immediately before use. After establishment of the whole-cell configuration, cellular capacitance was minimized using the analog compensation available on the amplifier. Series resistance was < 10 MΩ and compensated to > 85% in all the experiments. Data were filtered at 1 kHz (8-pole Bessel) and digitized at 10 kHz with a Digidata 1320 interface (Molecular Devices). For current-voltage (*I*-*V*) relationship studies, the membrane potential was held at –110 mV and cells were depolarized from –80 to 20 mV in 10-mV increments. For steady-state inactivation studies, the membrane potential was depolarized by test pulses to 0 mV for Ca_v_2.2, after 3.6 s conditioning pre-pulses ranging from –110 to 0 mV. Individual sweeps were separated by 12 s.

### Electrophysiological properties of hNa_v_1.7 channels

For patch-clamp analysis, HEK293 cells stably expressing hNa_v_1.7 (Na_v_α co-expressed with the Na_v_ β_1_ subunit; Scottish Biomedical) were cultured following the manufacturer's guidelines. The cells were removed from the culture at 70% confluence using Stempro Accutase (Life Technologies, Australia) and resuspended to 1×10^6^ cells/mL in Ex-Cell ACF CHO Medium (Life Technologies, Australia) supplemented with 25 mM HEPES (Sigma-Aldrich, Australia) and 1 × Glutamax (Life Technologies, Australia) before being transferred to the QPatch QStirrer (Sophion Bioscience, Ballerup, Denmark) and allowed to recover for 30 min before use. The external solution for all Na_v_ recordings contained (in mM): NaCl (140), KCl (4), CaCl_2_ (2), MgCl_2_ (1), HEPES (10), TEA-Cl (20) and glucose (10) containing 0.1% BSA. The pH was adjusted to 7.4 with NaOH and osmolarity adjusted to 315 mOsm with sucrose. The intracellular solution consisted of (in mM): CsF (140), EGTA/CsOH (1/5), HEPES (10) and NaCl (10) adjusted to pH 7.4 with CsOH and 320 mOsm with sucrose. The whole-cell patch-clamp experiments were performed at room temperature on a QPatch 16 automated electrophysiology platform (Sophion Bioscience) using 16-channel planar patch-chip plates (QPlates) with a patch hole diameter of 1 μm and resistance of 2 ± 0.02 MΩ. The cell positioning and sealing parameters were: positioning pressure –60 mbar, minimum seal resistance 0.1 GΩ, holding potential –100 mV and holding pressure –20 mbar. Whole-cell currents were filtered at 5 kHz (8-pole Bessel) and digitized at 25 kHz. A P4 online leak-subtraction protocol was used with non-leak-subtracted currents acquired in parallel.

The cells expressing hNa_**v**_1.7 were maintained with a holding potential of –100 mV and *I*-*V* relationships determined using a family of 500 ms conditioning pulses from –120 mV to +70 mV in 5-mV steps, followed by depolarization to 0 mV to assess the voltage dependence of fast inactivation. Each sweep was separated by 20 s to allow complete recovery from inactivation. State-dependence was assessed after a 10 min compound incubation to ensure steady-state inhibition for each concentration. In order to assess compound activity at the partially inactivated/open state, a series of 10 × 50 ms depolarizations to 0 mV were measured after corresponding conditioning pulses to –55 mV for 8 s with a 50 ms recovery before the test pulse, and cycled over a 12 s period for recovery of inactivation. To assess possible interaction with the voltage sensor domain, a triple-pulse protocol was used, comprising two steps to 0 mV for 50 ms separated by a strong depolarization depolarisation step to 200 mV for 50 ms, with 20 ms recovery to the –100 mV holding potential between each step.

### Electrophysiology data analysis

The Ca_v_ channel electrophysiology data were analyzed using Clampfit 9.2 (Molecular Devices). Curves were fitted using Origin 7.5 software (Northampton, MA, USA). Electrophysiology data for Na_v_ channels were assessed using the Sophion QPatch Assay Software v5.0, with curves fitted using GraphPad Prism v5.0. *I*-*V* relationships were fitted with using a modified Boltzmann equation: *I* = [*G*_max_*(*V*_*m*_–*E*_*rev*_)]/[1+exp(*V*_*a*_–*V*_*m*_)/*k*_a_)], where *V*_*m*_ is the test potential, *V*_*a*_ is the half-activation potential, *E*_*rev*_ is the reversal potential, *G*_*max*_ is the maximum slope conductance, and *k*_*a*_ reflects the slope of the activation curve. The voltage dependence of steady-state inactivation was calculated by dividing the amplitude of the test current (*I*) by the maximal current elicited (*I*_*o*_). Steady-state inactivation curves were fitted using the Boltzmann equation: *I/I*_*o*_ = 1/(1+exp((*V*_*m*_–*V*_*h*_)/_k_)), where *V*_*h*_ is the half-inactivation potential, *k* is the slope factor and *V*_*m*_ is the holding voltage. Statistical significance was determined by paired or unpaired Student’s *t*-tests and one-way or repeated measures ANOVA for n ≥ 6 independent experiments, followed by Dunnett’s post-hoc test. Differences were considered significant if p *<* 0.05. Data are expressed as mean ± SEM.

### Animal behaviour assessment

All the experiments involving animals were conducted according to the International Association for the Study of Pain Guidelines for the Use of Animals in Research (http://www.iasp-pain.org), in agreement with the Animal Care and Protection Regulation Qld (2012), and the Australian Code of Practice for the Care and Use of Animals for Scientific Purposes, 8th edition (2013) (http://www.nhmrc.gov.au). Ethics approval was obtained from the University of Queensland Institutional Animal Ethics Committee. C57BL/6J, an inbred mouse strain widely used in biomedical research [28], was used in all animal tests. Prior to experimentation, adult C57BL/6J male mice (5–8 weeks of age, average weight 20–25 g) were housed in groups of 2–4 under 12 h light-dark cycle, with free access to standard rodent chow and water. After experiments animals were euthanized by CO_2_ asphyxiation. All efforts were made to reduce the number and minimize the suffering of animals.

To test the analgesic potential of Cd1a, while avoiding potential off-target related side effects produced by systemic administration, we used a mouse model of Na_v_1.7-mediated pain, as previously described [29], based on intraplantar injection of the α/β scorpion toxin OD1 [30]. Briefly, a concentration of OD1 found to induce pain-like behaviour in mice (300 nM/40 μL diluted in sterile saline/0.1% BSA) [29] was injected subcutaneously into the subplantar surface of the left hind paw of mice (intraplantar, i.pl.) under light isoflurane anaesthesia (3%). Control animals received OD1 (300 nM) and treated animals received Cd1a (0.1 nM–10 μM/40 μL), Cm1a, Cm1b or tetrodotoxin (TTX) (1 μM/40 μL co-injected with OD1). Immediately after injection, the mice were placed into polyvinyl boxes (10 × 10 × 10 cm) and, after recovery from isoflurane, animals were monitored with a video camera placed under the boxes. Spontaneous pain behaviour (paw flinches, shakes and licks) and side-effects were visually assessed over 10–30 min by an observer blinded to all groups. Motor performance was assessed using the Parallel Rod Floor Test, with each peptide administered i.pl. 5 min before the testing. Distance travelled (m) and number of foot slips were recorded using ANY-Maze software (Stoelting Co., version 4.70, Wood Dale, IL, USA). The ataxia index was calculated by dividing the number of foot slips by the distance travelled.

Data were fitted using GraphPad Prism v5.0 and are presented as mean ± SEM (*n* = 6–12 mice). Statistical significance was determined using analysis of variance (ANOVA) with a Dunnett’s post-test, with significance defined as p < 0.05.

### Blowfly toxicity assay

To determine Cd1a activity in insects we used a previously reported method [31] with some modifications. Synthetic Cd1a was dissolved in insect saline and injected into the ventro-lateral thoracic region of adult sheep blowflies (*Lucilia cuprina*) with an average mass between 26.9 and 29.3 mg. A 1.0 mL Terumo Insulin syringe (BD Ultra-Fine, Terumo Medical Corporation, MD, USA) with a fixed 29 G needle fitted to an Arnold hand micro-applicator (Burkard Manufacturing Co. Ltd., England) was used to inject a maximum volume of 2 μL per fly. All flies were individually housed in 2-mL tubes and paralytic activity and lethality were determined at 1 h and 24 h post-injection. A total of three tests were carried out and for each test seven doses of Cd1a (*n* = 10 flies per dose) and the appropriate control (insect saline; *n* = 20 flies each) were used. PD_50_ values were calculated as described previously [31] using Prism 6.

### Materials

TTX, Cm1a and Cm1b were purchased from Alomone Labs, Jerusalem, Israel. OD1 and CVID were synthesized as previously described [30, 32, 33]. All the other chemicals were purchased from Sigma-Aldrich Australia, unless otherwise indicated.

## Results

### Discovery, synthesis and biochemical analysis of Cd1a

**Fig 1:**
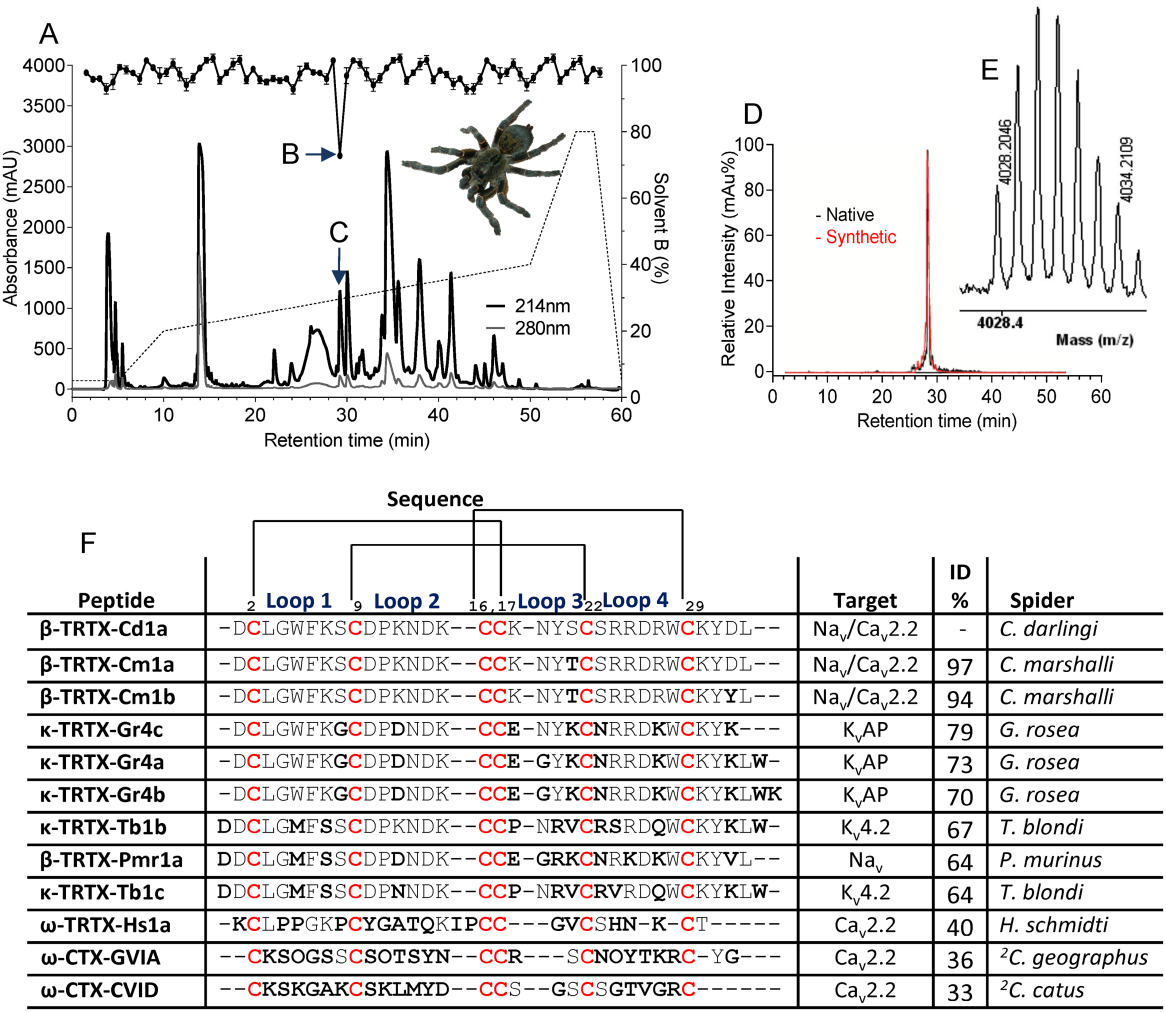
Assay-guided isolation of Cd1a. Assay-guided fractionation of Cd1a from *C*. *darlingi* venom. (**A**) Crude venom (0.5 mg/100 μL) was injected onto a RP-HPLC column (Vydac C18) and fractionated using a linear gradient of solvent B: 5% for 5 min, 5–20% for another 5 min and 20–40% over 40 min reaching 80% at 50–60 min. Fraction corresponding to Cd1a eluted with a retention time of 28 min corresponding to ~30% solvent B (indicated by vertical arrow). This fraction fully inhibited KCl/CaCl_2_-evoked Ca_v_2.2 responses (indicated by black circles over the chromatogram and a horizontal arrow) in SH-SY5Y cells, as measured by fluorescence-based Ca^2+^ imaging assays. (**D**) Analytical HPLC traces for native (black) and synthetic (red) Cd1a show identical retention time and peak width. MALDI-TOF analyses. The observed mass of native Cd1a (M+H: 4028.2 Da) was consistent with that predicted from the amidated Edman-derived amino acid sequence. (**F**) Sequence alignment of Cd1a with similar spider peptides and peptidic Ca_v_2.2 inhibitors (ω-toxins) from spider and cone snail venoms. Sequences were manually aligned. Non-conserved residues (compared to Cd1a) are highlighted in bold while the conserved cysteine framework is shown in red. The location of the four intercystine loops and the disulfide connectivity pattern are shown above the alignment. Abbreviations: Spider/cone snail *genus*: *C*. = *Ceratogyrus*, *G*. = *Grammostola*, *T*. = *Theraphosa*, *P*. = *Pterinochilus*, *H*. = *Haplopelma*, ^2^*C*. = *Conus*.

To find novel Ca_v_2.2 inhibitors, we screened 60 spider venoms using fluorimetric assays and SH-SY5Y cells expressing hCa_v_2.2. Approximately 30% of the venoms tested inhibited ≥ 50% of the hCa_v_2.2 responses (S1 Table), confirming spider venoms are rich sources of Ca_v_2.2 inhibitors. Venom from the African spider *C*. *darlingi* (spider shown in Fig 1A) fully inhibited hCa_v_2.2 (estimated 37 ng/μL). A search on ArachnoServer, a database listing spider venom toxins [20], indicated that there was no other Ca_v_2.2 inhibitor discovered from *C*. *darlingi* venom and thus, we selected this venom for fractionation. A single fraction eluting as a sharp peak at ~30% solvent B and ~99% purity (based on the HPLC profile) inhibited hCa_v_2.2 (Fig 1A). MALDI-TOF MS analysis indicated that the active fraction was dominated by a single mass (charged monoisotopic [M+H]^+^) of 4028.2 Da (Fig 1B). Edman sequencing revealed a 33-residue peptide sequence (DCLGWFKSCDPKNDKCCKNYSCSRRDRWCKYDL-NH_2_), with MALDI-TOF analysis indicating that the peptide was C-terminally amidated (~ –1 Da difference from the mass calculated for the free acid form of the Edman-derived sequence).

Cd1a was chemically synthesised using stepwise SPPS. A prominent disulfide bond isomer purified by HPLC to > 97% homogeneity had identical molecular mass to, and co-eluted with, native Cd1a (Fig 1B). Synthetic Cd1a inhibited Ca_v_2.2, further confirming that the synthetic peptide was correctly folded.

### Sequence homology studies

Surprisingly, BLAST searches on ArachnoServer database [20, 34] revealed that Cd1a was poorly similar to other Ca_v_2.2 inhibitors. The most similar Ca_v_ inhibitor isolated from a spider venom was ω-TRTX-Hs1a (Huwentoxin-X) [35], which shares only 40% identity with Cd1a. Cd1a lacks most of the functional residues shown to be important for Ca_v_2.2 block by ω-conotoxin CVID (Fig 1C), the most selective Ca_v_2.2 inhibitor described to date [33].

Interestingly, Cd1a is orthologous to β-TRTX-Cm1a (97% identity) and β-TRTX-Cm1b (94% identity), two peptides known to be Na_v_ channel inhibitors, isolated from the venom of the closely related tarantula spider *C*. *marshalli* (see Fig 1C) [36]. Cm1a and Cm1b belong to the NaSpTx Family 1 of spider toxins [19, 20]. Peptides from NaSpTx family 1 exhibit a conserved cysteine connectivity (C1–C4, C2–C5, C3–C6) and adopt an inhibitor cystine knot (ICK) fold [37, 38]. ICK frameworks have an antiparallel β-sheet stabilised by a cystine knot, formed by three disulfide bridges [36, 37].

### Cd1a inhibited human Ca_v_ channels

**Fig 2:**
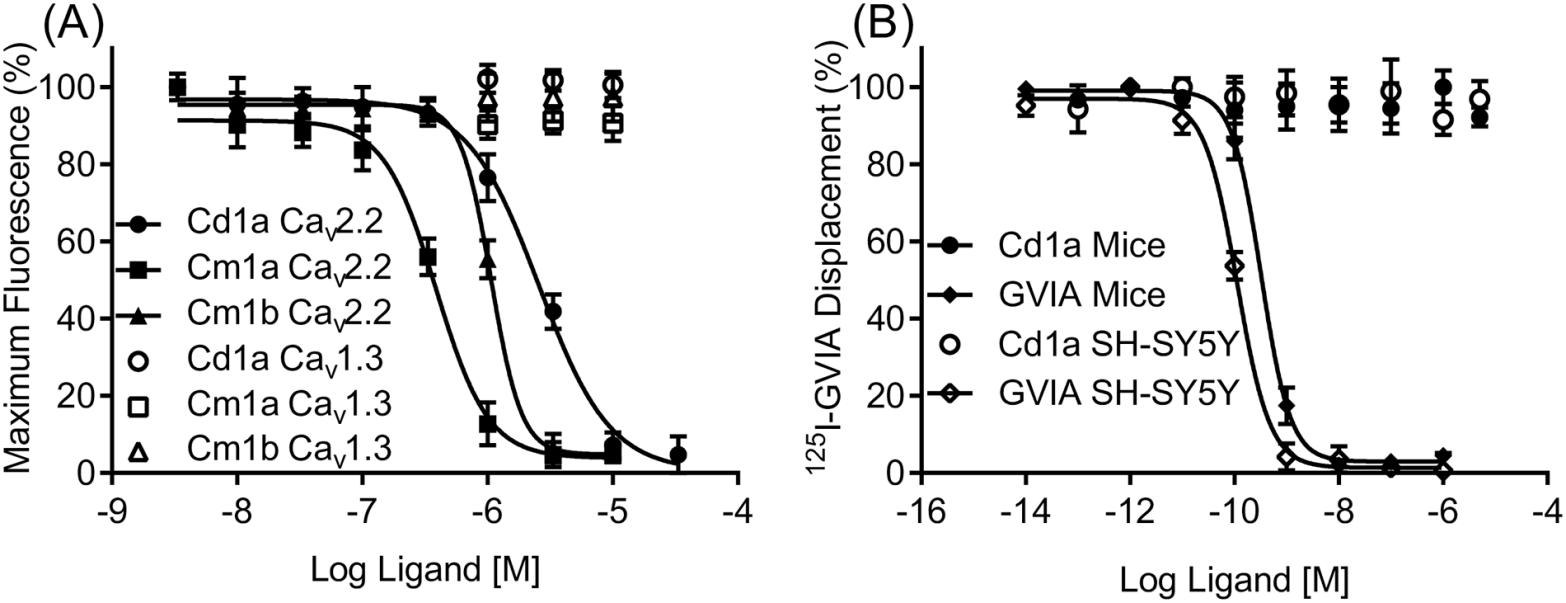
Activity of Cd1a, Cm1a and Cm1b on human Ca_V_ channels. Representative concentration-response curves for Cd1a and Cm1a—b obtained using fluorescence-based Ca^2+^ imaging assays. (**A**) Cd1a and Cm1a—b fully inhibited KCl/CaCl_2_-activated hCa_v_2.2 responses in SH-SY5Y cells (IC_50_ values (μM): Cd1a 2.6 ± 0.93, Cm1a 0.4 ± 0.2 and Cm1b 0.08 ± 0.02) but not Ca_v_1.3 or Ca_v_3.1 (IC_50_ > 10 μM). (**B**) Sigmoidal concentration response curves representing one single ^125^I-GVIA binding assay experiment. Unlabeled GVIA fully displaced ^125^I-GVIA from SH-SY5Y cell membranes (IC_50_ = 0.18 ± 0.01 μM) and mouse brain membranes (IC_50_ = 0.27 ± 0.01 μM), whereas Cd1a was unable to displace ^125^I-GVIA at concentrations up to 10 μM. Data points are mean ± S.E.M (*n* = 3–6 replicates).

We assessed Cd1a activity on native hCa_v_1.3, hCa_v_2.2 and hCa_v_3.1 channels expressed in SH-SY5Y cells [21], and compared with Cm1a and Cm1b activity. Cd1a, Cm1a and Cm1b fully inhibited ω-conotoxin-sensitive hCa_v_2.2 responses, in SH-SY5Y cells (IC_50_ values (μM): Cd1a 2.6 ± 0.93 μM; Cm1a 0.4 ± 0.2; and Cm1b 1.1 ± 0.6; Fig 2A). These results contrast with a previous report on patch clamp electrophysiology data, which indicated that Cm1a and Cm1b were inactive at HVA calcium currents of sensory neurons [36]. Cd1a, Cm1a and Cm1b were inactive on nifedipine-sensitive Ca_v_1.3 and mibefradil/pimozide-sensitive Ca_v_3.1 (Cd1a tested up to 30 μM and Cm1a and Cm1b 10 μM). Despite a tendency to some variability in potencies, the Ca_v_2.2 activity of the three peptides were not statistically different (two-way Anova).

### Cd1a binding on Ca_v_2.2 did not completely overlap the GVIA binding site

We used radiolabeled GVIA (^125^I-GVIA) in competitive binding studies to investigate the Cd1a mode of action. ω-Conotoxin GVIA is known to bind to Ca_v_2.2 channel α-subunit pore, in a region localized within the external EF hand motif of domain III S5–S6 (see S1 Fig) [26, 39]. Surprisingly, whereas unlabeled GVIA (used as a control) fully displaced radiolabeled ^125^I-GVIA from both human SH-SY5Y (IC_50_ of 0.18 ± 0.01 nM) and mouse brain cell membranes (IC_50_ of 0.27 ± 0.01 nM) (Fig 2B) with potencies similar to previous described [21, 24], Cd1a (10 μM) did not displace ^125^I-GVIA from these membranes, indicating that the Cd1a binding site does not overlap the GVIA binding site on Ca_v_2.2 channels.

### Cd1a did not act as a classical gating modifier toxin on Ca_v_2.2 channels

**Fig 3:**
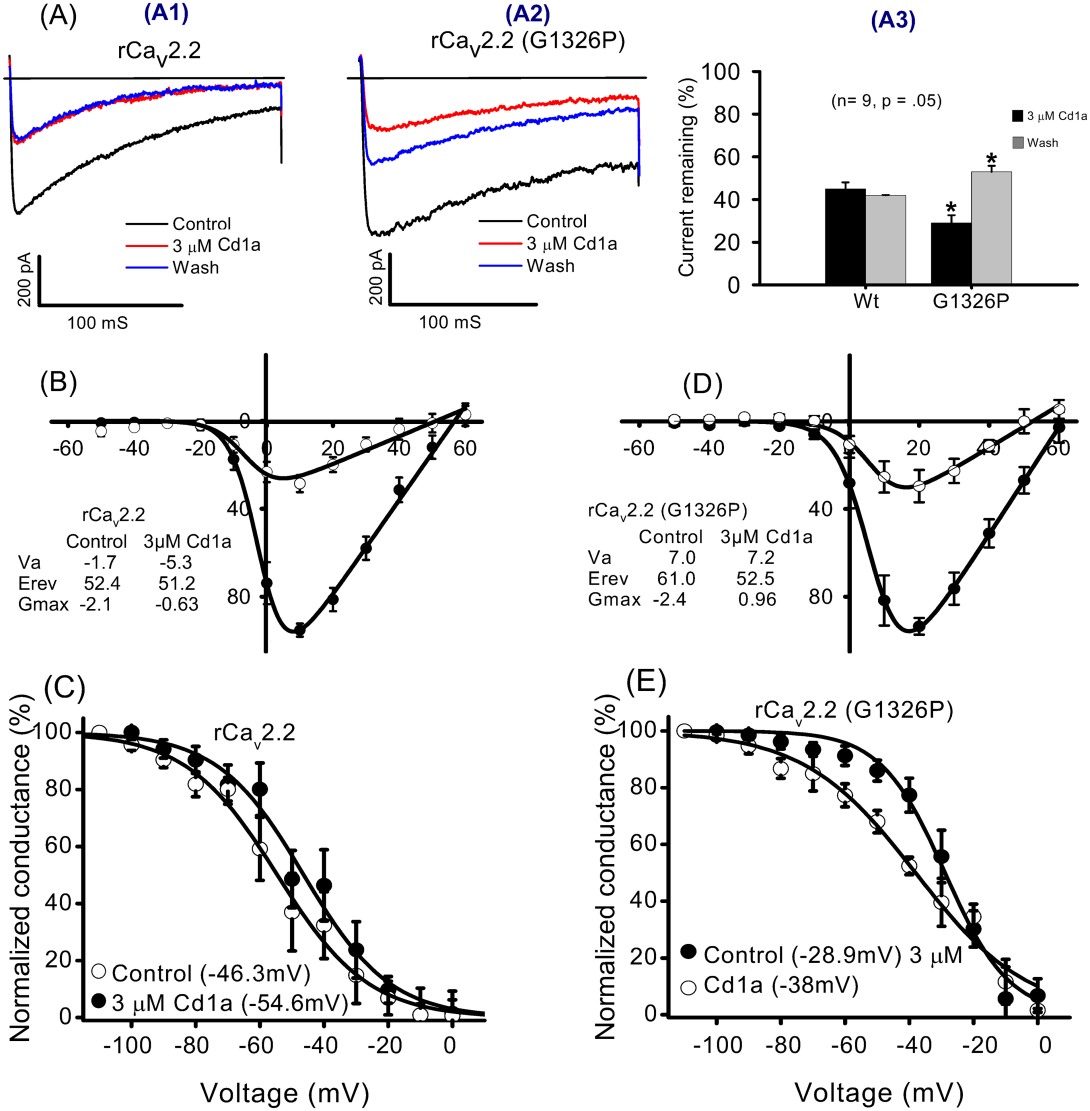
Effect of Cd1a on Ca_v_2.2 measured using patch-clamp electrophysiology. Representative whole-cell current traces and current-voltage relations recorded from wild-type (Wt) rCa_v_2.2 and mutant G1326P rCa_v_2.2 (α_1B_ + auxiliary β_1b_ and α_2_δ) expressed in tsA cells. (**A**) Inset shows the effects on current and reversibility before Cd1a addition (control, black), after (3 μM, red) Cd1a addition and after Cd1a wash-off for 5 min (blue). (**A1**) Cd1a (3 μM) inhibited Wt rCa_v_2.2 currents by ~50% and this effect was not reversed by a 5 min wash-off period. (**A2**) Inhibition was significantly higher for the mutant G1326P but the interaction was partly reversed by a 5 min wash-off. (**A3**) Histogram summarizing the percentage of remaining currents recorded from Wt and G1326P mutant channels before addition of 3 μM Cd1a and after wash-off. (**B**–**E**) Steady state activation (V_a_) and inactivation (V_h_) curves before (control) and after Cd1a (3 μM) addition. Cd1a had no significant effect on V_a_ of native (**B**) or mutant (**D**) channel (V_a_ of native: control = –1.7 mV, after Cd1a = –5.3 mV; V_a_ of mutant G1326P: control = 7.0 mV, after Cd1a = 7.2 mV). Cd1a had no effect on the reversal potential of the native channel, but induced a small shift on the mutant (E_rev_ of native: control = 52.4 mV, after Cd1a = 51.2 mV; E_rev_ G1326P: control = 61 mV, after Cd1a = 52.5 mV). (**C, E**) Inactivation curves for Ca_v_2.2 Wt (**C**) and mutant (**E**). Note a significant leftward shift in V_h_ in the presence of 3 μM Cd1a for both native and mutant (V_h_ for native: control = –46.3 mV, after Cd1a = –54.6 mV; V_h_ control of G1326P: –28.9 mV, after Cd1a = –38 mV). Data are mean ± S.E.M (*n* = 9 replicates).

The Cd1a effect was investigated on Ba^2+^ currents measured using whole-cell voltage-clamp electrophysiology on rCa_v_2.2 (Ca_v_α_1B_, Ca_v_β_1b_ + Ca_v_α_2_δ) expressed in tsA cells. Cd1a (3 μM) inhibited the currents by ~50% (*n* = 9) and there was no recovery from block during a 5 min wash-off period (Fig 3A). The reversal potential and half-activation voltage for Cd1a-treated cells (E_rev_ = +51.2 mV, V_a_ = –5.3 mV) were not significantly different from untreated cells (E_rev_ = +52.4 mV, V_a_ = –1.7 mV) (Fig 3B). In contrast, Cd1a produced a statistically significant hyperpolarizing shift in the half-inactivation potential of ~8 mV (V_h_ control = –46 mV; V_h_ Cd1a = –54.6 mV (Fig 3C). The absence of a measurable effect on the voltage dependence of activation under our conditions indicates. Cd1a has a unique mode of action at Ca_v_2.2 channels, differing from that of classical gating modifier toxins, such as ω-grammotoxin SIA and ω-IVA [39].

### Cd1a affected mutant rCa_v_2.2-G1326P function

We tested the ability of Cd1a to inhibit rCa_v_2.2 carrying a G1326P mutation (Ca_v_2.2-G1326P), which lies on the large extracellular loop region of the Ca_v_2.2 α subunit. This mutation results in localized structural disruption within the domain III S5–H5 region associated with the external EF hand motif within domain III S5–S6 [26, 40, 41] that alters ω-conotoxin GVIA and MVIIA affinity and reversibility [41], associating this location with the binding site for these toxins. Whereas wild-type rCa_v_2.2 channels showed no recovery from Cd1a-evoked block, Cd1a inhibition of the mutant Ca_v_2.2-G1326P channel was reversed following a 5 min washout (Fig 3A). In addition, Cd1a induced a significant shift in E_rev_ of the mutant channel (control = 61.0 mV, Cd1a = 52.5 mV) in contrast to its lack of effect at the wild-type channel. The voltage dependence of activation of the mutant, like the native rCa_v_2.2, was unaffected (V_a_ control = 7.0 mV, V_a_ Cd1a = 7.2 mV), whereas the voltage dependence of inactivation underwent a small but significant shift (V_h_ control = –28.9 mV; V_h_ Cd1a –38 mV (Fig 3D and 3E). The combined data suggest that, despite no overlap in binding sites demonstrated by the inability of Cd1a to displace GVIA in biochemical studies, there is some overlap in the molecular determinants of channel interactions between these two toxins. However, more extensive channel mutagenesis is required to fully define the Cd1a binding site.

### Cd1a activity on 8 heterologously expressed hNa_v_ channels

**Fig 4:**
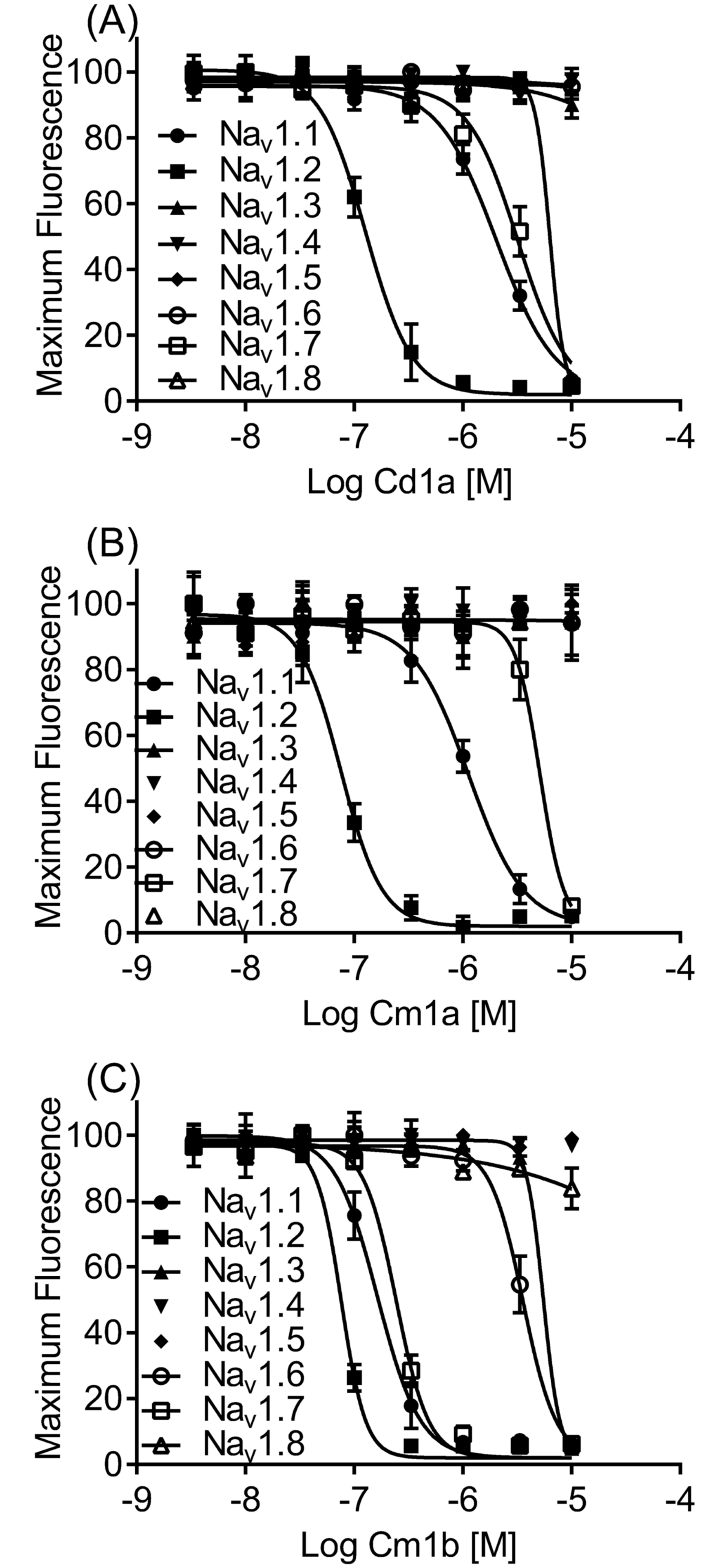
Activity of Cd1a and Cm1a—b on human Na_V_ channels. Representative concentration-response curves for Cd1a (**A**), Cm1a (**B**), and Cm1b (**C**) on hNa_v_ channels heterogously expressed in HEK293 cells. Data are from the FLIPR fluorimetric membrane potential assays. Cd1a and Cm1a—b inhibited a range of Na_v_ isoforms with variable potency (see IC_50_ values for the three toxins on Na_v_1.1– Na_v_1.8 in Table 1). Data points are mean ± S.E.M (*n* = 3–6 replicates).

Due to the high similarity between Cd1a and the Na_v_ channel inhibitors Cm1a and Cm1b, we assessed the effect of Cd1a on hNa_v_1.1–1.8 channels heterologously expressed in HEK293 cells using fluorimetric assays, and compared with the effects of Cm1a and Cm1b using the same assays. Overall, Cd1a activity was not statistically different to Cm1a and Cm1b (two-way Anova), with each peptide inhibiting Na_v_1.1, Na_v_1.2 and Na_v_1.7 (IC_50_ values in Fig 4A, Table 1). Cd1a (30 μM) and Cm1a (10 μM) (Fig 4B) were inactive at Na_v_1.3–Na_v_1.6 (Fig 4A–4C, Table 1), whereas Cm1b (Fig 4C) was also inactive at Na_v_1.4–Na_v_1.5 (up to 10 μM), but inhibited hNa_v_1.3 and Na_v_1.6. Cd1a was the only peptide with activity at Na_v_1.8 (Fig 4A, Table 1) in our assays, in contrast with previous report on rat clones using patch clamp electrophysiology [36], where 2 μM Cm1a and Cm1b inhibited 55 and 40% of Na_v_1.8 current, respectively.

**Table 1:** Cd1a, Cm1a and Cm1b activity on Na_v_1.1–1.8 channels.

Na_v_ subtype	Cd1a (IC_50_, μM)*	Cm1a (IC_50_, μM)	Cm1b (IC_50_, μM)
**Na**_**v**_**1.1**	2.18 ± 0.69	1.06 ± 0.32	0.17 ± 0.11
**Na**_**v**_**1.2**	0.13 ± 0.04	0.07 ± 0.22	0.08 ± 0.02
**Na**_**v**_**1.3**	> 30	> 10	5.57 ± 0.04
**Na**_**v**_**1.4**	> 30	> 10	> 10
**Na**_**v**_**1.5**	> 30	> 10	> 10
**Na**_**v**_**1.6**	> 30	> 10	3.99 ± 1.60
**Na**_**v**_**1.7**	3.34 ± 1.56	5.12 ± 2.85	0.23 ± 0.13
**Na**_**v**_**1.8**	6.92 ± 3.44	> 10	> 10

*Data are expressed as mean ± SEM.

### The Cd1a mode of action on hNa_v_1.7

**Fig 5:**
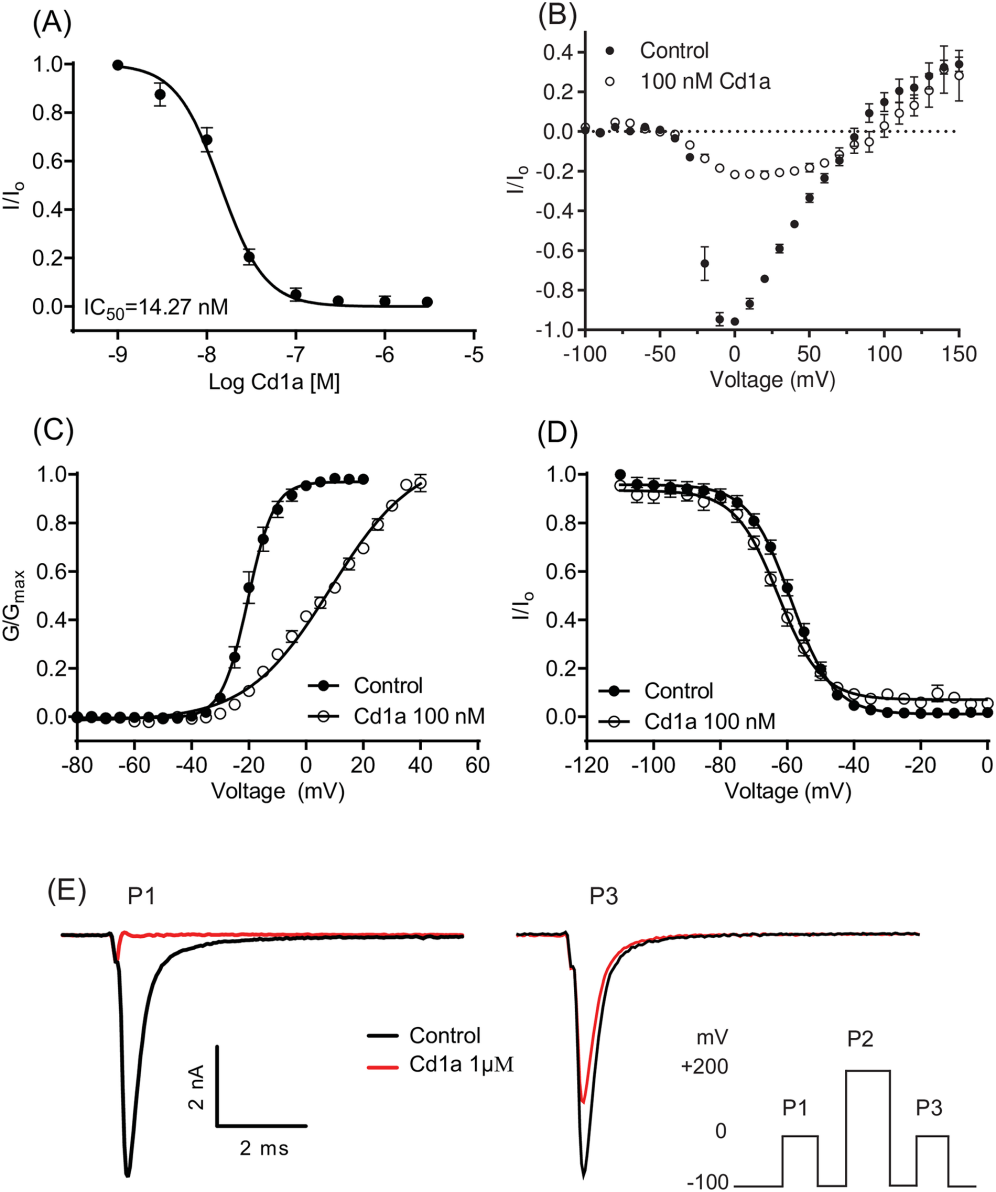
Electrophysiological analysis of Cd1a mode-of-action on Na_v_1.7. Data were acquired using patch-clamp of HEK293 cells stably expressing hNa_v_1.7. (**A**) Representative concentration-response curve for Cd1a inhibition of hNa_v_1.7 (IC_50_ 16.0 ± 3.0 nM). (**B**) Current-voltage relationship (*I-V*) curve determined using a holding potential of –100 mV and a family of 500-ms conditioning pulses from –120 mV to +70 mV in 5-mV steps, followed by a depolarization to 0 mV. Cd1a (100 nM) blocks a large percentage of the inward Na_v_1.7 currents but not the outward currents. (**C**) Cd1a (100 nM) shifted V_a_ by 29 mV (V_a_ control = –20.35 ± 0.3 mV; V_a_ Cd1a = 8.57 ± 1.03 mV) (**D**) without significantly affecting V_h_ (V_h_ Control = –58.9 ± 0.38; V_h_ Cd1a = –62.69 ± 0.48 mV). (**E**) Inset (bottom right corner) shows the effect of a triple-pulse protocol comprising two steps to 0 mV for 50 ms (P1 and P3) separated by a strong depolarization step to +200 mV for 50 ms, with 20 ms recovery (P2), using a holding potential of –100 mV between each step. Cd1a (1 μM) fully inhibited peak current in P1. Current inhibition was partially reversed in P3 using the positive depolarizing pulse protocol suggesting interaction of Cd1a with one of the Na_v_1.7 voltage sensors. Data points are expressed as mean ± S.E.M (*n* = 9–13 replicates).

We assessed the Cd1a effect on hNa_v_1.7 channels stably expressed in HEK293 cells using planar patch-clamp electrophysiology. Cd1a inhibited the hNa_v_1.7 peak current in a concentration-dependent manner, with an IC_50_ in the low nanomolar range (16.0 ± 3.0 nM, *n* = 10; Fig 5A). An *I-V* family of conditioning pulses and their corresponding *G-V* curves were used to determine the effect of Cd1a on the voltage dependence of activation (V_a_), while depolarization to 0 mV following a conditioning pulse was used to assess its effects on the steady-state inactivation (V_h_) (Fig 5B). Interestingly, Cd1a (100 nM) induced a significant 29 mV depolarizing shift in V_a_ (control –20.4 ± 0.3 mV; Cd1a 8.6 ± 1.0 mV, *n* = 11) (Fig 5C). Furthermore, at 100 nM Cd1a induced a small 3.8 mV hyperpolarizing shift of the voltage dependence of inactivation (V_h_) of hNa_v_1.7 (control –58.9 ± 0.4 mV; Cd1a –62.7 ± 0.5 mV, *n* = 9) (Fig 5D).

We used a triple pulse protocol previously described [36, 42] to identify if Cd1a interacted with the Na_v_1.7 voltage sensor domains. Under control conditions there was no significant change between a first (P1) and third (P3) pulses separated by a strong positive pulse (P2) (*Insert* Fig 5E). Cd1a at 1 μM fully inhibited P1 as expected, however, the P3 current was not fully inhibited, indicating partial relief of block by P2. This suggests that Cd1a interacts with one or more voltage sensor domain of hNa_v_1.7 channels.

### Cd1a is analgesic in a Na_v_1.7 mouse model of peripheral pain

**Fig 6:**
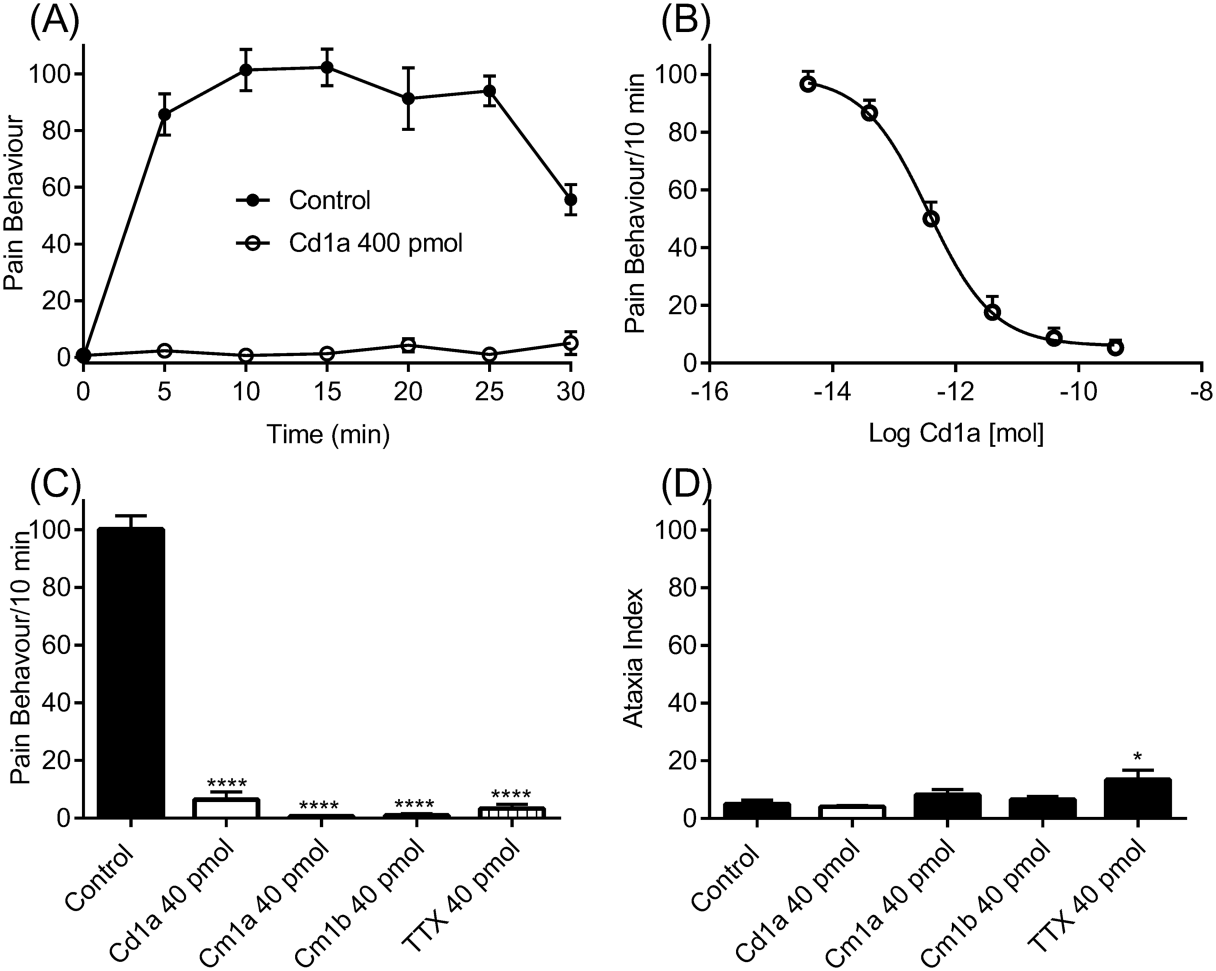
Cd1a reverses pain behaviour in mice. Cd1a reversed pain behaviours in a mouse model of peripheral spontaneous pain generated by intraplantar injection of the Na_v_1.7 activator OD1 [29,30,32,43]. (**A**) OD1 (300 nM/40 μL; control) evoked spontaneous pain behaviours (paw flinches, shakes and licks) in mice for up to 30 min (time observed) and these behaviours were fully reversed by Cd1a (400 pmol). (**B**) Reversal of pain behaviours by Cd1a was concentration dependent (IC_50_ = 0.36 ± 0.12 pmol). (**C**) Comparison of Cd1a, Cm1a, Cm1b and TTX reversal of OD1-evoked pain behaviours. (**D**) Ataxia index (number of foot slips/meters travelled) after intraplantar delivery of Cd1a, Cm1a, Cm1b and TTX. TTX significantly increased the ataxia index (*p *<* 0.05) whereas Cd1a, Cm1a, Cm1b had no significant effect compared to vehicle control (saline/0.1% BSA). Histogram values are mean ± S.E.M (*n* = 6–12 mice).

Intraplantar injection of the scorpion toxin OD1 evokes spontaneous pain behaviours (paw licks, shakes and flinches) in mice (Fig 6A), an effect mostly mediated by Na_v_1.7 channels [29]. Intraplantar injections of Cd1a (400 pmol) completely reversed OD1-evoked spontaneous pain behaviours for at least 30 min (Fig 6A). The Cd1a effect was concentration dependent (IC_50_ = 0.36 ± 0.12 pmol) (Fig 6B). For comparison, we tested Cm1a, Cm1b and TTX in the same mouse model. At a dose that all peptides with activity at Na_V_1.7 reversed spontaneous pain behaviours (40 pmol), TTX caused sedation, and reduced motor coordination (Fig 6C and 6D) in all mice, whereas Cd1a, Cm1a and Cm1b did not produce any apparent side effects.

### Cd1a reversibly paralysed sheep blowfly

**Fig 7:**
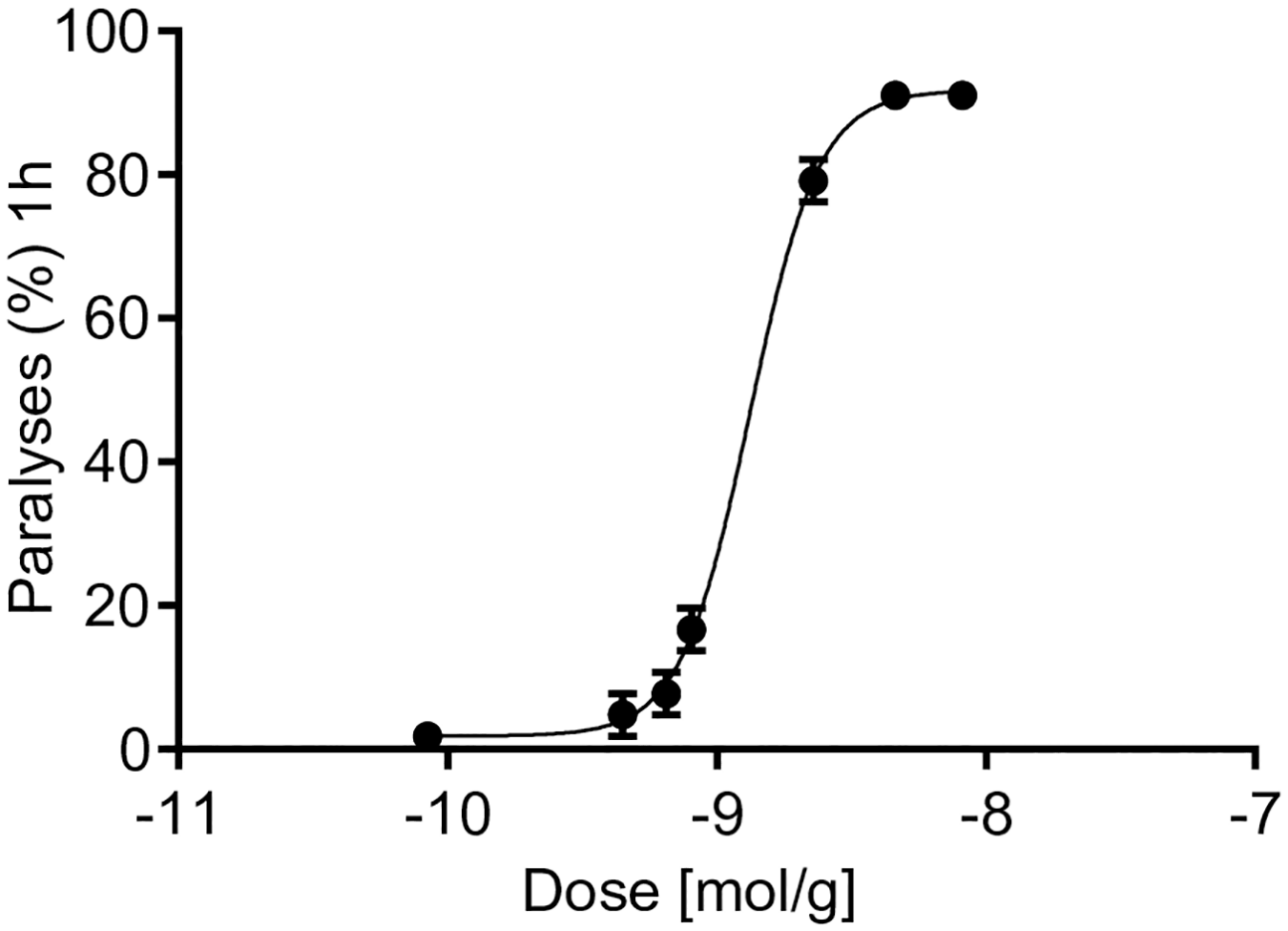
Paralytic effects of Cd1a in sheep blowflies. The insecticidal activity of Cd1a was determined by injection of Cd1a into the ventro-lateral thoracic region of adult sheep blowflies (*Lucilia cuprina*) with average masses between 26.9 and 29.3 mg. Paralytic effects were determined 1 h after injection (PD_50_ 1318 pmol/g). No signs of paralysis or lethality were observed 24 hours post-injection (data not shown).

Cd1a produced paralytic effects in adult sheep blowfly, with a PD_50_ measured 1 hour after injection of 1318 ± 58 pmol/g (Fig 7). Remarkably, however, all flies fully recovered from the initial paralytic effects induced by Cd1a at doses up to 8.17 nmol/g.

## Discussion

We describe the discovery and characterization of Cd1a, the first Na_v_/Ca_v_ inhibitor peptide reported from the African theraphosid spider *C*. *darlingi*. Cd1a belongs to NaSpTx family 1 [19], a class of promiscuous toxins that can modulate a range of ion channels, including Na_v_, Ca_v_, K_v_, mechanosensitive and proton-gated ion channels. Peptides from NaSpTx family 1 share an ICK structural motif that typically provides resistance to heat denaturation and proteolysis [37, 44], features that are potentially advantageous for drug development. Interestingly, Cd1a inhibits important peripheral nociceptive targets, including Na_v_1.7, Na_v_1.8 and Ca_v_2.2, but not cardiac Ca_v_ and Na_v_ channels (Ca_v_1.3, Ca_v_3.1 and Na_v_1.5), or Na_v_ channels found in skeletal muscle (Na_v_1.4) or nodes of Ranvier in motor nerves (Na_v_1.6). The Cd1a selectivity profile is consistent with its analgesic efficacy and lack of side effects at maximal efficacious doses in a mouse model of peripheral pain shown in this work.

Cd1a has little sequence similarity to other venom peptides that inhibit Ca_v_ channels, likely reflecting its relatively low potency against Ca_v_2.2 (IC_50_ of ~3 μM at rat and human Ca_v_2.2). Like Cd1a, the related peptides Cm1a and Cm1b from *C*. *marshalli* [36] are known Na_v_ inhibitors and have little sequence similarity to Ca_v_ inhibitors. However we found for the first time that these peptides inhibit hCa_v_2.2 with moderate potency, but were inactive at hCa_v_1.3 and hCa_v_3.1. Our results contrasted with a previous report on patch clamp electrophysiology, which indicated that Cm1a and Cm1b were inactive at HVA calcium currents of sensory neurons [36]. However Cm1a and Cm1b were tested at a maximum concentration of 100 nM [36], thus at a higher concentration it’s conceivable that these peptides would also be active in their system. Unlike classical Ca_v_ channel gating modifier toxins, Cd1a didn’t affect activation gating of rCa_v_2.2 but instead induced a small shift in the voltage-dependence of inactivation, suggesting that at this channel Cd1a doesn’t act as a typical gating modifier toxin. Cd1a activity was enhanced at the rCa_v_2.2α1B-G1326P mutant channel while channel inhibition became partially reversible, indicating possible partial overlap with the binding site for ω-conotoxins. In addition, Cd1a caused a small change in reversal potential of the mutant channel but not the wild-type, while channel inhibition became partially reversible, indicating binding near the ion permeation/selectivity pathway. However, competitive binding studies revealed that the Cd1a binding does not completely overlap with the GVIA binding site on mutant Ca_v_2.2. Thus, we suggest a possible allosteric coupling between the Cd1a and GVIA binding site that is introduced by the rCa_v_2.2α1B-G1326P mutation. Residues in the outer vestibule of the channel pore may allosterically affect Cd1a binding and its ability to interact with the inactivated state of the channel. Precisely how Cd1a influences the voltage dependence of inactivation remains to be determined, but it is possible that the toxin interacts with one or more of the S6 regions, which have been linked to fast inactivation [45].

The high level of homology between Cd1a and the Na_v_ inhibitor peptides Cm1a and Cm1b suggested that Na_v_ channels may be the primary high-affinity target for Cd1a. Indeed, in electrophysiological studies Cd1a was ~200-fold more potent at recombinant hNa_v_1.7 than rCa_v_2.2, inhibiting hNa_v_1.7 with an IC_50_ of ~15 nM. Whereas we have previously shown that species differences can lead to discrepancies in potencies [21], the IC_50_ for Cd1a at human and rat Ca_v_2.2 were similar in both in patch clamp and fluorescence assays (~3 μM), confirming the primary target for Cd1a is Na_v_1.7.

Using a FLIPR membrane potential dye assay, we identified that Cd1a, Cm1a and Cm1b were similarly active across a range of hNa_v_ channels, including Na_v_1.1–1.2 and Na_v_1.7. However, Cd1a potency at Na_v_1.7 was lower using the FLIPR assays than when using patch clamp electrophysiology (~200-fold), consistent with previous reports [46]. In addition we could not detect Na_v_1.8 activity for Cm1a and Cm1b, in contrast with previous report [36]. Although an explanation for these differences remains to be elucidated, a number of factors may affect between-assay peptide potency. These include different expression systems and β subunit combinations, species differences, influence of membrane potential dye, and the requirement for channel activation by veratridine in fluorescence assays. Nonetheless, fluorescence-based assays allow high-throughput screening and rapid assessment of pharmacology. While the rank order of potency across Na_v_ isoforms is typically conserved, differences in potencies between patch clamp electrophysiology (‘the gold standard method for assessing ion-channel function’) and fluorescence based assays have been described [46, 47, 48].

Although Cd1a didn’t affect the steady-state voltage parameters of activation of rat Ca_v_2.2, the human Na_v_1.7 inhibition was driven by a depolarising shift in V_a_. This effect on Na_v_1.7 is consistent with gating modifying activity through one or more of the voltage sensor domains (VSD) [36, 39, 42]. A forced outward movement of one or more Na_v_ voltage sensor domains has been achieved with strong positive depolarizing pulses, releasing gating modifier toxins from the voltage sensor domains [36, 42] and leading to recovery from toxin inhibition. In support, the strong positive-depolarising pulse we used in our patch clamp experiments enhanced recovery from Cd1a inhibition by ~70% and inhibition of inward but not outward current were observed, similar to other gating modifier toxins [36, 42].

Due to its pharmacology profiling and mode of action, with the highest activity at Na_v_ channels, Cd1a was named β-theraphotoxin-Cd1a (or β-TRTX-Cd1a as abbreviation), based on the rational nomenclature devised for spider toxins [49]. While the exact site of Cd1a interaction with Na_v_ channels remains to be elucidated, spider-venom peptides with similar modes of action have been found to bind to VSDs DI—DIII but not to DIV, which appears to influence inactivation rather than activation [50–52].

We assessed the analgesic potential of Cd1a by examining its ability to reverse spontaneous pain induced by intraplantar injection of the Na_v_1.7 channel activator OD1 [29, 30, 43]. Remarkably, Cd1a was able to completely reverse the OD1-induced nociceptive behaviour in mice, with no apparent off-target related side effects at the highest dose tested and no motor side effects. These results contrasted with TTX, which also fully reversed the OD1-induced pain behaviour at the same dose, but caused sedation and motor incoordination, suggesting off-target neuronal and/or skeletal muscle effects associated with its inhibition of Na_v_1.1–1.4 and/or Na_v_1.6 [53]. Consistent with the lack of side effects seen for Cd1a in mice, Cd1a was inactive on cardiac and peripherally expressed Ca_v_1.3, Ca_v_2.1 Na_v_1.4, Na_v_1.5 and Na_v_1.6 (30 μM). Cd1a was potent at centrally expressed Na_v_1.1–1.2, however, it is unlikely to cross the blood-brain barrier and interact with these channels centrally [9].

In future, Cd1a needs to be tested in other more conventional animal models of pain, such as the inflammation and neuropathic pain models, to confirm its usefulness as a peripheral analgesic lead, and to provide information tools to compare Cd1a with peptides from other animal venoms.

Whereas the analgesic effect of Cd1a might be useful as a therapeutic lead, we wondered about the ecological significance of Cd1a. Pain-inducing venom components for defensive purposes have been previously reported from tarantula venoms [31], but the analgesic activity of Cd1a does neither endow the spiders with an evolutionary advantage nor support a potential defensive purpose. Hence, we assumed that it might be used for predation and with insects and other invertebrates being the main prey of tarantulas [54] we tested Cd1a in a blowfly toxicity assay. The fact that Cd1a induced paralysis in blowflies supports a potential role for Cd1a in predation.

In summary, we have isolated and pharmacologically characterized Cd1a, a novel peptide inhibitor that showed insecticidal effects and acts on the anti-nociceptive targets Ca_v_2.2, Na_v_1.7 and Na_v_1.8. Cd1a additionally inhibits the central Na_v_1.1–1.2, but not peripheral off-target channels such as Ca_v_1.3 and Ca_v_2.1 or Na_v_1.4–1.6. Interestingly, Cd1a acts at Ca_v_2.2 and Na_v_1.7 with distinct modes of action, inhibiting Ca_v_2.2 activity affecting near the pore region, but not overlapping the ω-conotoxin binding site, interfering with inactivation with no apparent effects on the activation gating of the channel. Conversely, at Na_v_1.7 Cd1a acts as a typical gating modifier toxin, interacting with one or more of the voltage sensor domains and changing the gating properties of this channel. The primary structure of Cd1a strongly suggests that it will fold into an ICK motif that is expected to provide a high level of chemical, thermal and biological stability. Thus, Cd1a may be a useful lead for development of a peripherally acting analgesic. In future studies it will be important to examine Cd1a analgesic potential in a wider range of pain models to confirm its usefulness as an analgesic lead.

## Supporting information

S1 Table List of spiders tested for Ca_v_2.2 activity. (DOCX)

S1 Fig Topology of the calcium channel α-subunit pore. Proposed transmembrane topology of the calcium channel α1 subunit indicating the location of the putative EF hand motif in IIIS5-H5 where G1326P is located.
(TIF)
