# Supplementary material for: Discovery and mode of action of a novel analgesic β-toxin from the African spider Ceratogyrus darlingi
Source: PLoS One. 2017 Sep 7;12(9):e0182848. doi: 10.1371/journal.pone.0182848 (PMC5589098; doi:10.1371/journal.pone.0182848)
Supplement: S1 Table — (DOCX) [file pone.0182848.s001.docx]

**Table SI. List of spiders tested for Ca_v_2.2 activity.**

| **Genus** | **Species** | **Family** | **Continent** | **Active** |
| --- | --- | --- | --- | --- |
| *Acanthoscurria* | *geniculata* | Theraphosidae | America | N |
| *Atrax* | *robustus* | Hexathelidae | Australia | Y |
| *Badumna* | *insignis* | Desidae | Australia | N |
| *Brachypelma* | *albopilosum* | Theraphosidae | America | Y |
| *Brachypelma* | *annitha* | Theraphosidae | America | N |
| *Brachypelma* | *boehmei* | Theraphosidae | America | N |
| *Brachypelma* | *smithi* | Theraphosidae | America | N |
| *Ceratogyrus* | *darlingi* | Theraphosidae | Africa | Y |
| *Ceratogyrus* | *brachycephalus* | Theraphosidae | Africa | Y |
| *Ceratogyrus* | *marshalli* | Theraphosidae | Africa | Y |
| *Cyclosternum* | *spec.(Colombia)* | Theraphosidae | America | N |
| *Cyriopagopus* | *schioedtei* | Theraphosidae | Asia | Y |
| *Davus* | *fasciatus* | Theraphosidae | America | N |
| *Ephebopus* | *cyanognathus* | Theraphosidae | America | N |
| *Ephebopus* | *murinus* | Theraphosidae | America | N |
| *Ephebopus* | *rufescens* | Theraphosidae | America | N |
| *Eriophora* | *transmarina* | Araneidae | Australia | N |
| *Eupalaestrus* | *campestratus* | Theraphosidae | America | N |
| *Grammostola* | *rosea* | Theraphosidae | America | Y |
| *Haplopelma* | *doriae* | Theraphosidae | Asia | Y |
| *Haplopelma* | *lividum* | Theraphosidae | Asia | Y |
| *Haplopelma* | *minax* | Theraphosidae | Asia | N |
| *Haplopelma* | *schmidti* | Theraphosidae | Asia | N |
| *Heteroscodra* | *maculata* | Theraphosidae | Africa | N |
| *Holothele* | *spec.(Venezuela)* | Theraphosidae | America | N |
| *Holothele* | *spec.(Peru)* | Theraphosidae | America | N |
| *Megadolomedes* | *australianus* | Pisauridae | Australia | N |
| *Monocentropus* | *balfouri* | Theraphosidae | Africa | N |
| *Nephila* | *plumipes* | Nephilidae | Australia | N |
| *Nhandu* | *tripepii* | Theraphosidae | America | N |
| *Orphnaecus* | *spec.("treedweller", Philippines)* | Theraphosidae | Asia | N |
| *Orphnaecus* | *spec.(Maanghit-Cave, Philippines)* | Theraphosidae | Asia | N |
| *Orphnaecus* | *spec.(Sibaliw, Philippines)* | Theraphosidae | Asia | N |
| *Pamphobeteus* | *antinous* | Theraphosidae | America | N |
| *Pamphobeteus* | *nigricolor* | Theraphosidae | America | Y |
| *Pamphobeteus* | *spec.(Colombia)* | Theraphosidae | America | Y |
| *Phoneutria* | *nigriventer* | Ctenidae | America | N |
| *Phormictopus* | *cancerides* | Theraphosidae | America | N |
| *Phormictopus* | *cochleasvorax* | Theraphosidae | America | Y |
| *Plesiopelma* | *spec.(Bolivia)* | Theraphosidae | America | Y |
| *Poecilotheria* | *fasciata* | Theraphosidae | Asia | N |
| *Poecilotheria* | *ornata* | Theraphosidae | Asia | Y |
| *Poecilotheria* | *regalis* | Theraphosidae | Asia | N |
| *Poecilotheria* | *striata* | Theraphosidae | Asia | N |
| *Poecilotheria* | *vittata* | Theraphosidae | Asia | Y |
| *Psalmopoeus* | *irminia* | Theraphosidae | America | N |
| *Psalmopoeus* | *pulcher* | Theraphosidae | America | N |
| *Pseudhapalopus* | *spinulopalpus* | Theraphosidae | America | N |
| *Pterinochilus* | *murinus* | Theraphosidae | Africa | N |
| *Schismatothele* | *spec.(Colombia)* | Theraphosidae | America | N |
| *Selenocosmia* | *javanensis sumatrana* | Theraphosidae | Asia | Y |
| *Selenocosmia* | *spec.(Borneo)* | Theraphosidae | Asia | N |
| *Selenotholus* | *cf foelschei* | Theraphosidae | Australia | Y |
| *[Sparassidae]* | *spec.(Fraser Island, Australia)* | Sparassidae | Australia | N |
| *Stromatopelma* | *calceatum* | Theraphosidae | Africa | N |
| *Tapinauchenius* | *cupreus* | Theraphosidae | America | N |
| *Tapinauchenius* | *elenae* | Theraphosidae | America | Y |
| *Theraphosa* | *apophysis* | Theraphosidae | America | N |
| *Theraphosa* | *blondi* | Theraphosidae | America | N |
| *Thrixopelma* | *pruriens* | Theraphosidae | America | N |

Y= Yes (Active: at least 50% inhibition); N= No (Less than 50% inhibition).
