# Supplementary figures and images for: Discovery and mode of action of a novel analgesic β-toxin from the African spider Ceratogyrus darlingi
Source: PLoS One. 2017 Sep 7;12(9):e0182848. doi: 10.1371/journal.pone.0182848 (PMC5589098; doi:10.1371/journal.pone.0182848)

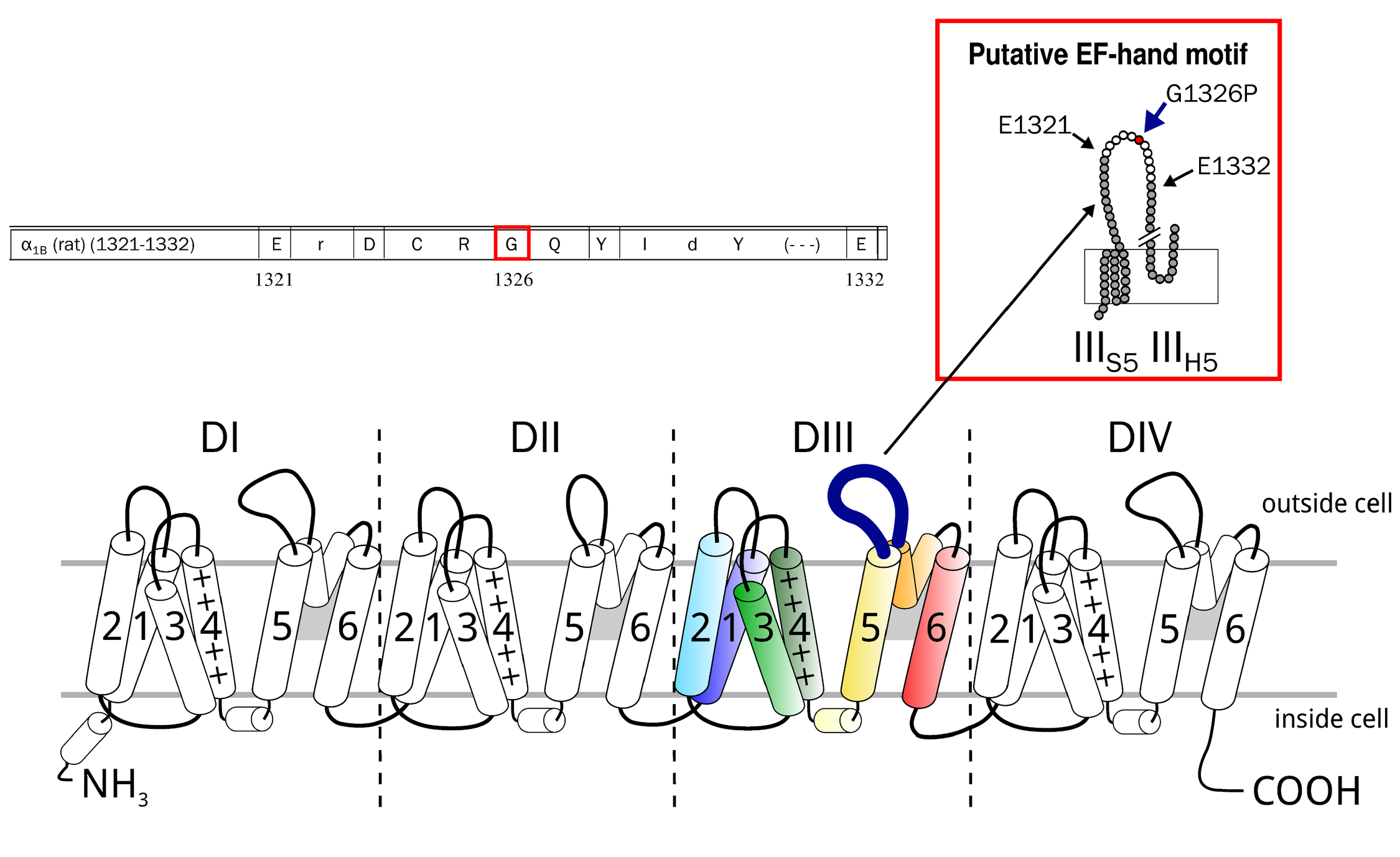

Supplement: S1 Fig — Proposed transmembrane topology of the calcium channel α1 subunit indicating the location of the putative EF hand motif in IIIS5-H5 where G1326P is located. (TIF) [file pone.0182848.s002.tif]
